# Mutual Interaction of Clinical Factors and Specific microRNAs to Predict Mild Cognitive Impairment in Patients Receiving Hemodialysis

**DOI:** 10.3390/cells9102303

**Published:** 2020-10-15

**Authors:** Jin-Bor Chen, Chiung-Chih Chang, Lung-Chih Li, Wen-Chin Lee, Chia-Ni Lin, Sung-Chou Li, Sin-Hua Moi, Cheng-Hong Yang

**Affiliations:** 1 Division of Nephrology, Department of Internal Medicine, Kaohsiung Chang Gung Memorial Hospital and Chang Gung University College of Medicine, Kaohsiung 833, Taiwan; r5239@cgmh.org.tw (L.-C.L.); leewenchin@gmail.com (W.-C.L.); 2 Department of Neurology, Kaohsiung Chang Gung Memorial Hospital and Chang Gung University College of Medicine, Kaohsiung 833, Taiwan; neur099@adm.cgmh.org.tw; 3 Department of Laboratory Medicine, Chang-Gung Memorial Hospital at Linkou Medical Center and Department of Medical Biotechnology and Laboratory Science, Chang Gung University, Taoyuan 33303, Taiwan; chianilin@cgmh.org.tw; 4 Clinical Genomics & Proteomics Core Lab, Department of Medical Research, Kaohsiung Chang Gung Memorial Hospital, Kaohsiung 833, Taiwan; pinus@cgmh.org.tw; 5 Center of Cancer Program Development, E-Da Cancer Hospital, I-Shou University, Kaohsiung 833, Taiwan; moi9009@gmail.com; 6 Department of Electronic Engineering, National Kaohsiung University of Science and Technology, Kaohsiung 833, Taiwan; chyang@cc.kuas.edu.tw

**Keywords:** mild cognitive impairment, hemodialysis unit, microRNAs

## Abstract

Cognitive impairment (CI) is not uncommon in dialysis patients. Various factors have been implicated. This study aims to examine mutual interaction of various clinical factors for CI in patients receiving hemodialysis. A total of 48 hemodialysis patients in outpatient clinic were recruited from 2015 to 2017. Demographics, circulating uremic toxin concentrations, miRNA concentrations, and nerve injury protein concentrations were collected. Clinical dementia rating (CDR) scores were used to stratify the functional scores of the patients. Receiver operating characteristic (ROC) analysis was used to evaluate diagnostic test performance for predicting dichotomous results, and cumulative ROC analysis was used to examine the combined contribution of clinical factors. CDR scale 0 included 15 patients (mean age, 59.1 years); CDR > 0.5 included 33 patients (mean age, 64.0 years). On cumulative ROC analysis, the major predictors of mild CI were hemoglobin, age, sex, homocysteine, neuron-specific enolase (NSE), and miR-486. The cumulative area under the curve (AUC) on combining hemoglobin, age, and miR-486 was the highest (0.897, 95% confidence interval 0.806–0.988). Two dichotomized variables reached 81.82% sensitivity and 86.67% specificity, with the likelihood ratio for positive and negative results being 6.14 and 0.21, respectively. In conclusion, hemoglobin, age, and miR-486 display high-degree combined effects on mild CI in patients receiving hemodialysis.

## 1. Introduction

Cognitive impairment (CI) has a high prevalence in chronic kidney disease (CKD), especially in elderly patients [1,2,3]. The clinical picture consists of cognitive slowing and executive, memory, and language deficits. The contributing factors include cerebral white matter disease [4,5], silent brain infarcts [6,7], demographic factors [8,9,10,11], vascular risk factors [12], uremic toxins [13], secondary hyperparathyroidism [14], and dialysis disequilibrium [15].

The CI observed in CKD influences not only daily life and ability to work but also results in longer hospitalization and higher risk for mortality [13,16,17]. In patients receiving hemodialysis exhibiting CI, the average time to death was 1.09 years and the hazard ratio (HR) for death was 1.87, which was higher than that observed in patients receiving hemodialysis with cardiac disease (HR, 1.28) or stroke (HR, 1.20) [13].

Although various factors may contribute to CI in patients with CKD, these factors are generally treated individually, without considering possible mutual interactions. Recently, receiver operating characteristic (ROC) analysis is being widely used to evaluate diagnostic test performance for predicting dichotomous results by comparing sensitivity and specificity [18,19]. Further, ROC analysis has been improved by simultaneously considering multiple factors, termed cumulative ROC analysis.

In the current study, we aimed to use ROC analysis to examine the contribution between demographics (age and sex), routine biochemistry variables, uremic toxins, and miRNAs for CI prediction in patients receiving hemodialysis. We also aimed to identify high risk factors through cumulative ROC analysis.

## 2. Materials and Methods

### 2.1. Participants

**Figure 1:**
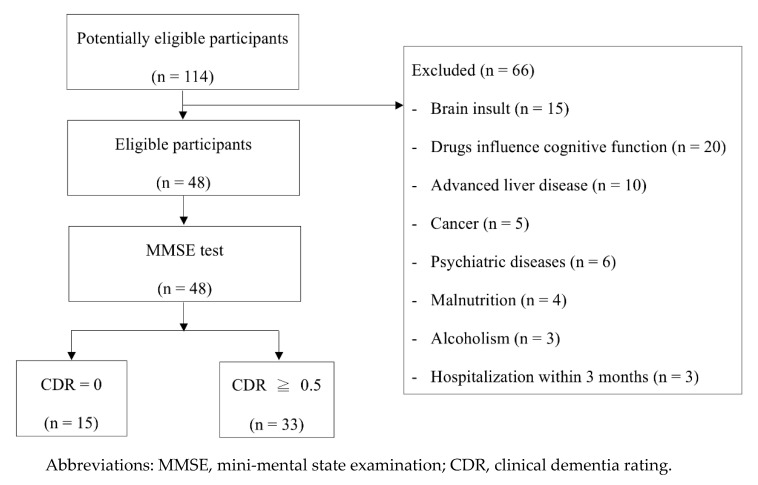
Participants flow diagram.

The study period was from 2015 to 2017. Patients who received regular outpatient hemodialysis at Kaohsiung Chang Gung Memorial Hospital in Taiwan were enrolled. The inclusion criteria were (1) age ≥ 18 years; (2) subjective memory complaints; and (3) ability to provide basic interview, meaning no aphasia. The exclusion criteria included (1) history of cerebral stroke or brain injury by nonmedical causes; (2) drug-usage history, which may influence cognitive function, including chemotherapy; (3) other comorbidities, which may affect cerebral function, e.g., liver cirrhosis, cancer, and psychiatric diseases; (4) malnutrition, serum albumin level <3.5 g/dL; (5) alcoholism; (6) pregnancy; (7) hospitalization within 3 months of enrollment; and (8) absence of caregiver for providing medical history. All of the participants underwent hemodialysis thrice weekly with dialyzers of surface area ≥2.0 m^2^ and bicarbonate-based dialysate. A total of 114 potentially eligible hemodialysis patients were screened. Finally, a total of 48 patients receiving hemodialysis were enrolled for analysis (Figure 1). The study protocol was approved by the Committee on Human Research at Kaohsiung Chang Gung Memorial Hospital (number: 104-2572B). Written informed consent was obtained from participants. The study was conducted in accordance with the Declaration of Helsinki.

### 2.2. Laboratory Measurement

Blood sampling was performed mid-week (Wednesday/Thursday) with fasting status for participants who had received hemodialysis. Blood test was conducted for hemoglobin (Hb) and uremic toxins. Uremic toxins included (1) small water-soluble solutes, such as blood urea nitrogen (BUN), creatinine (Cr), calcium (Ca), phosphorus (P), asymmetric dimethylarginine (ADMA), and 8-hydroxy-2-deoxyguanosine (8-OHdG); (2) protein-bound solutes, such as p-cresyl sulfate (PCS), indoxyl sulfate (IS), and homocysteine; and (3) medium-sized molecules: interleukin 1-β (IL-1β), interleukin 6 (IL-6), interleukin 18 (IL-18), tumor necrosis factor-α (TNF-α), intact parathyroid hormone (iPTH), and β2 microglobulin. Blood samples for BUN, Cr, Ca, P, and glutamate oxaloacetate transaminase (GOT) were analyzed using commercial kits and an autoanalyzer (Hitachi 7600-210, Hitachi Ltd., Tokyo, Japan). The PCS and IS were quantified using HPLC (Waters TQ-S, Milford, MA, USA).

iPTH was measured by chemiluminescent immunoassay (Siemens Healthcare Diagnostics Inc., Norwood, MA, USA). Measurements of ADMA, 8-OHdG, IL-1β, IL-6, IL-18, and TNF-α were performed using ELISA. β2 microglobulin level was measured using a turbidimetry method (Spaplus, The Binding site Group Ltd., Birmingham, UK).

### 2.3. Nerve-Injury Proteins

We measured 3 nerve-injury-related proteins in the blood using ELISA: neuron-specific enolase (NSE); heat shock protein (HSP) 70, and S100B.

### 2.4. Measurement of Serum miRNAs Levels

We selected candidate miRNAs from peripheral mononuclear cells using next-generation sequencing (NGS). The candidate miRNAs were selected if their blood levels were higher than 1.5-fold in patients receiving hemodialysis compared to healthy volunteers. Thus, 4 miRNAs were identified: miR-134, miR-182, miR-451, and miR-486.

### 2.5. Method of NGS

The collected RNA samples were first subject to quality examination with Bioanalyzer 2100 (Agilent) facility. The RNA samples with RIN (RNA integrity number, determined with Bioanalyzer 2100) value ≥ 8.0 were prepared with TruSeq small RNA preparation protocol (Illumina, San Diego, CA, US), followed by sequencing with a V3 150-cycle sequencing reagent on the MiSeq facility (Illumina) to generate 51-nt single-end reads. The generated NGS data were analyzed with miRSeq tool kit file:///D:/Documents/My_Papers/FINISHED/51_ADHD_miRNA_Diagnosis panel/SUBMISSION/Manuscript_R1.doc - _ENREF_1#_ENREF_1 [20] with default parameters to quantify the expressions of human miRNAs (miRBase 20).

### 2.6. Quantitative PCR for miRNAs

For quantitative PCR of the miRNAs, 10 ng of total RNA was converted into cDNA using the TaqMan MicroRNA Reverse Transcription kit (Thermo Fisher Scientific Applied Biosystems, Foster City, CA, USA). The expression of mature miR-134, miR-182, miR-451, miR-486, and U6 (internal control) was quantified using the commercially available TaqMan Universal Master Mix No Amp UNG (Applied Biosystems, Foster City, USA) in a 7500 Real-Time PCR System. The qRT-PCR was performed at 95 °C for 10 min, followed by 40 cycles of 95 °C for 15 s and 60 °C for 1 min. Expression levels of the miRNAs were normalized to that of the internal control, U6, using the equation log (2^−ΔCt^), where ΔCt = Ct target − U6.

### 2.7. Mini-Mental State Examination

General cognitive function was assessed using the Mini-Mental State Examination (MMSE); this test have been validated in the Chinese population [21,22]. We used clinical dementia rating (CDR) scores to stratify the functional scores of the patients [23]. The CDR rated the participant’s impairments in 6 categories—memory, orientation, judgment and problem solving, community affairs, home and hobbies, and personal care—on a 5-point scale (0, 0.5, 1, 2, and 3). Summarizing the impairment ratings, all of the participants were assigned a rating score of CDR 0, indicating no dementia, and 0.5, 1, 2, and 3, indicating questionable, mild, moderate, and severe dementia, respectively. The necessary information was obtained through interview of the patient and reliable informant (e.g., family member) by one qualified reviewer based on CDR assessment protocol.

### 2.8. Statistical Analyses

The distribution of variables between the groups was summarized in terms of mean (standard deviation), median (interquartile range), or frequency (percentage). The difference between the groups was estimated using the independent two-sample *t*-test or *χ*^2^ test, as appropriate. In addition, the effect size of continuous variables between groups was estimated using Cohen’s *d* from the *t*-test. The dichotomous result of each variable was determined by individual ROC analysis. Higher individual area under the curve (AUC) represented better prediction performance for high CDR scores. The variables were then ranked by individual AUCs estimated from individual ROC analysis.

A cumulative ROC analysis was performed to detect the combined effects of the measurements used to predict a high CDR score. Positive changes in the cumulative AUCs were tracked along with the cumulated variables until the addition of other variables no longer increased the cumulative AUC. In cumulated ROC analysis, the likelihood ratio was used to assess high CDR scores in subjects with different cumulative scores. The LR+ (sensitivity/[1 − specificity]) represented the ratio of the probability of a positive test for subjects with high CDR scores to that of a positive test for subjects with low CDR scores. Therefore, the LR− ([1 − sensitivity]/specificity) represented the ratio of the probability of a negative test for subjects with high CDR scores to that of a negative test for subjects with low CDR scores. The 95% confidence interval (CI) for sensitivity, specificity, and likelihood ratios were computed.

Variables that contributed to positive changes for the highest cumulative AUC were selected for further analysis. The cumulative risk score for each subject was obtained by adding the risk score (1 or 0) of selected variables that contributed to the high CDR score. For each subject, the cumulative risk score represented the total number of risk factors. For instance, a score of 5 was interpreted as the presence of 5 risk factors that were most relevant to a high CDR score. In general, a limited sample size provides unusable results (e.g., wide confidence interval) and also generates an overfit model in regression analysis. Considering the abovementioned limitations, we conducted logistic regression and only included the most contribute factors for mild cognitive impairment in patients receiving hemodialysis derived from cumulative ROC results. A *p*-value of less than 0.05 was considered statistically significant. All statistical analyses were performed using STATA version 11.0.

## 3. Results

### Baseline Characteristics

The dialysis vintage in the study cohort was 119 ± 78 months. CDR scale 0 included 15 patients (mean age, 59.1 years; 5 men and 10 women); CDR ≥ 0.5 included 33 patients (mean age, 64.0 years; 18 men and 15 women; CDR = 0.5, 32 patients; CDR = 2, 1 patient). There were no significant differences in the blood levels of biochemical variables, small water-soluble solutes, protein-bound solutes, medium-sized molecules, and molecular markers of nerve injury, except that the BUN level was higher in patients with CDR 0 (75.00 mg/dL vs. 59.36 mg/dL; *t*-test, *p* = 0.009; Cohen’s *d* 0.88) and the Hb level was higher in patients with CDR ≥ 0.5 (10.83 g/dL vs. 9.92 g/dL; *t*-test, *p* = 0.010; Cohen’s *d* −0.97). The candidate miRNA analysis did not show significant difference in the blood levels of miR-134, miR-182, miR-451, and miR-486 (Table 1).

AUC was calculated for individual clinical and laboratory factors for CI prediction in patients receiving hemodialysis.

Table 2 presents the dichotomous results for various single demographic and laboratory factors listed in Table 1. None of the factors showed acceptable performance (AUC ≥ 0.7), except Hb (AUC = 0.792) and age (AUC = 0.708).

Table 3 shows the cumulative top-ranked clinical factors for predicting CI in patients receiving hemodialysis. The cumulative AUC for the combination of cumulative top-ranked clinical factors was calculated using the AUC for different combinations of factors, where the factors were added individually in the descending order of individual AUCs (Table 2). The highest score was obtained for a combination of three top-ranked clinical factors: Hb, age, and miR-486 (cumulative AUC = 0.897, 95% CI = 0.806–0.988). The second combination involved four top-ranked clinical factors: Hb, age, miR-486, and sex (cumulative AUC = 0.874, 95% CI = 0.768–0.981). Combinations of other top-ranked clinical factors showed a descending order of cumulative AUCs. Accordingly, Hb, age, sex, miR-486, homocysteine, and NSE were the predominant factors for predicting CI in patients receiving hemodialysis. Further, we calculated sensitivity and specificity for these dichotomized variables. Two out of the three top-ranked variables (Hb, age, and miR-486) showed a highest Youden’s index (68.49%) and correctly classified percentage (83.33%) than others (Table 4). The sensitivity was 81.82% and the specificity was 86.67%, classified with a likelihood ratio for positive (LR+) and negative (LR−) results being 6.14 and 0.21, respectively. The relationship between cumulated risk score and selected variables (Hb, age, and miR-486) are summarized in Table 5 by interpreting the proportion of patients in each cumulated risk score strata. The patients with cumulated risk score 1 had the highest proportion in miR-486 ≥ 32.68 (66.7%).

The information of distribution of MMSE in different CDR groups is shown in Supplementary Table S1. The results of cut-off point of cumulated risk score identified by ROC analysis for MMSE category derived from CDR dichotomous stratification is shown in Supplementary Table S2. Two out of the three top-ranked variables (Hb, age, and miR-486) showed the highest Youden’s index (24.17%) and correctly classified percentage (62.50%) than others. The sensitivity was 72% and the specificity was 52.17%, classified with a likelihood ratio for positive (LR+) and negative (LR−) results being 1.51 and 0.54, respectively. This relationship is shown in Supplementary Figure S1. This result indicated that CDR stratification was superior to MMSE stratification in examining association of clinical factors with cognitive impairment in our participants.

The cumulative ROC results indicated that Hb, age, and miR-486 exerted major contribution; hence, we included these variables for logistic regression analysis (Table 6). The univariate analysis indicated the association for each of the included variables for mild cognitive impairment, whereas the multivariate analysis considered the association simultaneously. Both univariate and multivariate analysis indicate the Hb (univariate: OR (95% CI) = 2.29 (1.13–4.64), *p* = 0.022; multivariate: OR (95% CI) = 2.74 (1.13–6.67), *p* = 0.026) and miR-486 (univariate: 4.24 (1.14–15.79), *p* = 0.031; multivariate: OR (95% CI) = 7.54 (1.47–38.6), *p* = 0.015) were significantly associated with mild cognitive impairment. Afterward, the 2-order interaction analysis, including Hb*Age OR (95% CI) = 1.01 (1.001–1.01), *p* = 0.019), Hb*miR-486 (OR (95% CI) = 1.17 (1.0–1.32), *p* = 0.016) and age*miR-486 (OR (95% CI) = 1.03 (1.004–1.05), *p* = 0.019) were given. The results indicated that all three combinations in 2-order interaction level were significantly associated with mild cognitive impairment in hemodialysis patients. Unfortunately, the 3-order interaction analysis were omitted due to the limited sample size and current regression algorithm. Overall, the 2-order interaction results were consistent with the cumulative ROC findings, which indicated that hemoglobin, age, and miR-486 were associated with mild cognitive impairment in patients receiving hemodialysis under certain interaction effects.

## 4. Discussion

One of the risk factors for CI is CKD. The potential causes involve vascular and neurodegenerative mechanisms [24]. The implicated factors range from traditional/nontraditional ones to direct neuronal toxicity due to uremic toxins [24]. However, the combined effect of these factors on CI in CKD remains unknown. Phenotype presentation of a disease is the consequence of combined effect of various molecules and signaling pathways in the organism. Therefore, we applied ROC analyses to examine the combined effect of plausible clinical factors for CI prediction in patients receiving hemodialysis. Based on baseline comparison, we did not find significant difference in the proposed factors between CI and non-CI participants. We also selected four candidate miRNAs—miR-134, miR-182, miR-451, and miR-486—using next-generation sequencing to compare CI and non-CI participants. The individual levels of these miRNAs did not show significant differences between the two cohorts. When we applied individual ROC analysis, Hb and age were the prominent factors, reaching AUC > 0.7. Further, we applied cumulative ROC analyses to examine the combined effect of the proposed factors. The combination of Hb, age, and miR-486 showed the best cumulative AUC for CI prediction in patients receiving hemodialysis. The other important combined factors included sex, homocysteine, and NSE (a nerve-injury protein). Thus, we propose a combined effect of clinical factors contributing to mild CI in patients receiving hemodialysis.

It has been reported that Hb shows a U-shaped association with cross-sectional cognitive function [25,26]. The plausible underlying mechanisms are inadequate cerebral oxygenation leading to impaired cerebral perfusion and cerebral function in low Hb concentrations [27]. In contrast, high Hb concentrations may represent hyperviscosity, hypovolemia, polycythemia vera, and pulmonary disease. These scenarios may lead to cerebral hypoxia and CI [25,28]. However, there is still no evidence to show that optimal Hb concentrations prevent CI in CKD. In the present study, we found that Hb showed the highest AUC on individual ROC analysis. Moreover, a cumulated ROC analysis exhibited an acceptable AUC for CI prediction in patients receiving hemodialysis. Based on our findings, we propose that future studies on an optimal Hb concentration for stratified age groups of subjects are necessary for prevention or treatment of CI.

Aging is a natural process in organisms. Complex sophisticated coordinated mechanisms among tissues and organs are involved in the aging process. Commonly, aging is characterized by the progressive decline in functions of tissues and organs. Eventually, aging contributes to the risk of disease occurrence. In the past decades, studies have investigated the molecular mechanisms of aging in different tissues [29,30,31,32]. Aging is well recognized as the greatest risk factor for the onset of age-related neurodegenerative diseases. Age-related alterations in the brain include cell adhesion molecules, neuronal activity, and neurotransmitter and neuromodulator action [30]. Our findings echo the aforementioned reports that aging contributes to CI in patients receiving dialysis. Nevertheless, this study is not sufficient to test our hypothesis for the plausible mechanism of age-related CI in patients receiving hemodialysis. An advanced study is warranted to explore the aging mechanism underlying the onset of CI in patients receiving dialysis.

Sex showed the fourth-highest AUC in our study. The cumulative AUC of Hb, age, sex, and miRNA-486 was 0.874. This finding indicates that sex plays an important role in CI of patients in Taiwan receiving dialysis. A nationwide survey in Taiwan showed that women had a higher prevalence than men for overall dementia and mild CI [33]. The exact explanation was not provided by the authors. In our study, we did not include economic status, educational levels, and comorbidities for comparison between men and women. Therefore, we can only conclude that sex contributes to CI in patients receiving hemodialysis. Further studies are needed to address the plausible mechanisms underlying the above observations.

We analyzed the contribution of several uremic toxins to CI in our study. Among them, homocysteine showed the highest AUC in individual ROC analysis. Homocysteine is a protein-bound solute and is commonly elevated in patients with CKD. On conversion to homocysteic acid, homocysteine can activate N-methyl-D-aspartate receptor, thus leading to a direct neurotoxic effect [34]. Homocysteine has been shown to be associated with faster rate of cognitive decline in a six-year follow-up study on elderly subjects [35]. Our study found homocysteine was one of the cumulated top-ranked factors for prediction of mild CI in patients receiving hemodialysis. The cumulative AUC was 0.835 when five factors (Hb, age, miR-486, sex, and homocysteine) were combined. However, the role of homocysteine in causing CI in patients receiving dialysis must be validated by a large-scale population study.

Circulating miRNAs have been reported to be biomarkers of mild CI. Two sets of miRNA pairs—miR-132 and miR-134 families—were shown to differentiate patients with mild CI from age-matched controls [36]. Next-generation sequencing for miRNA expression profiling is becoming a common technology for various diseases, including those affecting the kidney and brain [37,38]. We used this technology to profile miRNAs in patients receiving hemodialysis and identified four candidate miRNAs: miR-134, miR-182, miR-451, and miR-486. In our study, the plasma levels of individual miRNAs were not statistically different between patients with and without CI. Using cumulative ROC analyses, we found that the combination of Hb, age, and miR-486 showed an acceptable cumulative AUC (0.897). It seems that miR-486 plays a crucial regulatory role in CI in patients receiving hemodialysis. One of the miR-486 target genes, *GABRB3*, encodes gamma-aminobutyric acid receptor subunit beta-3 (GABRB3), which is a member of the ligand-gated ion channel family [39]. Gamma-aminobutyric acid is the major inhibitory neurotransmitter of the nervous system. The missense mutation of this gene may be associated with several nervous diseases, including epilepsy and autism [40]. However, the regulatory roles of miRNAs are complex. Individual miRNAs can target several mRNAs, and an mRNA can be targeted by more than one miRNA. This might, at least in part, explain why we could not find a role of the CI-related miR-134 [36] in patients receiving hemodialysis. The identified miRNAs for mild CI in our patients were different from those previously reported in subjects without CKD [36]. One concern is that serum miRNAs levels may be altered by hemodialysis procedure [41]. Because our participants received miRNAs examination prior to hemodialysis, we suppose that this concern may not influence our results. Currently, the evidence for differences in CI-related miRNA profiles between CKD and non-CKD cohorts is still lacking. Further studies on disease-specific miRNAs are required to clarify this issue.

A total of 152 uremic toxins have been detected, and these molecules have been shown to exert various negative effects, such as anorexia, cardiac failure, anemia, immune dysfunction, malnutrition, inflammation, and skin atrophy [24,42]. Uremic toxins have also been suspected to have a causal relationship with CI in CKD [43]. However, the impact and mechanism of action of each uremic toxin on cognition and cerebral nervous system in uremic state remains unknown. In our study, homocysteine and β2 microglobulin exhibited higher AUC than other uremic toxins. Guanidine compounds were found in the brain regions involved in cognition [44]. These compounds reportedly indirectly elevate serum homocysteine in cognitive disorders [45]. The definitive mechanisms of action of uremic toxins for the onset of CI in CKD require further investigation.

In our study, we measured blood levels of nerve-injury-related proteins, such as NSE, HSP 70, and S100B; NSE was one of the top-ranked variables on cumulative ROC analyses. It is a glycolytic isoenzyme expressed in the central and peripheral neurons and neuroendocrine cells. In rats, NSE immunofluorescence signal decreased in the affected neurons 2–10 days following axonal injury. There is accumulating evidence that the level of NSE in neurons serves as a marker of axon injury, regeneration, and target reinnervation [46,47,48]. Accordingly, we propose that NSE might be involved in the onset of CI in patients receiving hemodialysis.

The present study is subject to several limitations. Firstly, our study did not include a follow-up interval, and the cumulative ROC approach was limited to cross-sectional clinical data. This may ignore all possible combinations of complex interactions when the effect of time is considered. Secondly, our study did not include other possible factors affecting CI in patients receiving dialysis, such as economic and social status and comorbidities. Thirdly, we did our best to examine the implicated uremic toxin levels in our patients. However, there are several uremic toxins that we did not examine. Therefore, their contribution to CI in these patients was not investigated. Fourthly, the sample size was relatively small, and all the participants were Taiwanese. The possible effect of ethnicity cannot be ruled out in our study. Despite the above limitations, the strength of our study is in being the first investigation on a combination of specific clinical factors predicting mild CI in hemodialysis patients. This approach not only provides an alternative insight into the plausible mechanisms for CI but also a method for CI prediction in such patients.

## 5. Conclusions

The cumulative ROC analysis provides better AUC performance than individual ROC analysis for identifying factors for prediction of mild CI in patients receiving hemodialysis. Our proposed scoring system identified six clinical factors that displayed a high degree of combined effect for mild CI in patients receiving hemodialysis.

## Figures and Tables

**Table 1:** Baseline characteristics of study participants. (*n* = 48).

Variables	CDR = 0 (*n* = 15)		CDR ≥ 0.5 (*n* = 33)		*p*	Cohen’s *d*
	Mean	SD	Mean	SD		
Age (years)	59.1	±8.1	64.0	±9.5	0.092	−0.50
Gender (men, %)	5	33.33%	18	54.55%	0.173	
Education level					0.786	
No	0	0.00%	2	6.06%		
Primary school	5	33.33%	6	18.18%		
Elementary school	2	13.33%	4	12.12%		
High school	5	33.33%	11	33.33%		
Bachelor	2	13.33%	4	12.12%		
Unknown	1	6.67%	6	18.18%		
Laboratory measurement						
Kt/V	1.83	±0.39	1.82	±0.41	0.968	−0.001
Hb (g/dL)	9.92	±0.9	10.83	±1.15	0.010	−0.97
Albumin (g/dL)	3.80	±0.24	3.84	±0.36	0.685	−0.14
GOT (U/L) (median, interquartile range)	19	14–25	18.5	15–27.5	0.404	−0.26
Small water-soluble solutes						
ADMA (µmol/L) (median, interquartile range)	3.28	0.9–5.74	3.28	0.63–5.95	0.511	−0.23
8OHDG (ng/mL)	27.48	±0.67	27.07	±0.69	0.058	0.52
BUN (mg/dL)	75.00	±23.38	59.36	±15.9	0.009	0.88
Cr (mg/dL)	10.72	±4.29	9.31	±2.4	0.149	0.45
Ca (mg/dL)	9.51	±0.68	9.22	±1.77	0.492	−0.08
P (mg/dL)	5.76	±2.21	5.00	±0.45	0.661	0.50
K (mEq/L)	4.47	±0.84	4.36	±0.89	0.551	−0.21
Protein-bound solutes						
PCS (µg/mL)	25.14	±16.81	27.00	±21.18	0.766	−0.22
IS (µg/mL)	46.05	±20.94	38.85	±18.83	0.241	0.28
Homocysteine (µmol/mL)	26.82	±7.9	29.70	±10.15	0.351	−0.29
Middle molecules						
IL-1β(pg/mL) (median, interquartile range)	0.76	0.65–0.95	0.66	0.65–0.85	0.728	-0.13
IL-6(pg/mL) (median, interquartile range)	6.47	2.42–10.92	4.18	2.8–5.34	0.275	0.31
IL-18(ng/mL) (median, interquartile range)	111.99	90.13–166.47	108.64	90.78–140.18	0.122	0.50
TNF-α (pg/mL)	35.39	±10.98	34.88	±12.34	0.893	−0.03
iPTH (pg/dL)	260.7	108.7–715.8	190.85	121.55–507.4	0.392	0.18
Beta-2-microglobulin (µg/L) (median, interquartile range)	27,700	20,740–31,619.5	25,933.95	20,695.75–31,985.15	0.835	0.13
Molecular markers of nerve injury						
NSE (ng/mL) (median, interquartile range)	1556.04	936.89–2952.33	2418.23	1084.04–3520.94	0.896	−0.04
HSP 70 (ng/mL) (median, interquartile range)	0.14	0.08–0.16	0.13	0.11–0.14	0.294	−0.38
S100B (pg/mL) (median, interquartile range)	83.58	57.05–142.02	83.58	25.26–157.98	0.892	−0.13
MicroRNA						
miR-134 (median, interquartile range)	0.53	0.33–1.77	0.51	0.15–2.48	0.563	−0.17
miR-182 (median, interquartile range)	0.09	0.03–0.36	0.06	0.04–0.23	0.970	−0.04
miR-451 (median, interquartile range)	4.92	0.36–10.69	1.9	0.49–10.04	0.284	−0.32
miR-486 (median, interquartile range)	32.38	22.18–188.2	111.14	33.96–269.09	0.643	−0.07

*p*-value was estimated using independent two-sample *t*-test or *χ*^2^ test appropriately. Cohen’s *d* effect size corrected for uneven groups from *t*-test. Abbreviations: Hb, hemoglobin; GOT, glutamate oxaloacetate transaminase; ADMA, asymmetric dimethylarginine; 8OHDG, 8-hydroxy-2-deoxyguanosine; BUN, blood urea nitrogen, Cr, creatinine; Ca, calcium; P, phosphate; K, potassium; PCS, p-cresyl sulfate; IS, indoxyl sulfate; IL, interleukin; TNF-α, tumor necrosis factor-α; iPTH, intact parathyroid hormone; NSE, neuron-specific enolase; HSP, heat shock protein.

**Table 2:** Individual receiver operating characteristic (ROC) analysis of clinical measurements for clinical dementia rating (CDR) status.

Variable	AUC	Best Cutoff Value	Sensitivity	Specificity	Correctly Classified
Age	0.708	63	69.70%	66.67%	68.75%
Gender	0.606	Male	54.55%	66.67%	58.33%
Education level	0.517	Above Elementary school	70.37%	35.71%	58.54%
Laboratory measurement					
Kt/V	0.470	1.3	100.00%	13.33%	72.34%
Hb	0.792	10.7	66.67%	93.33%	75.00%
Albumin	0.503	4.08	28.13%	86.67%	46.81%
Small water-soluble solutes					
ADMA	0.476	1.85	60.61%	46.67%	56.25%
8OHDG	0.339	25.6	100.00%	0.00%	68.75%
BUN	0.297	31	96.97%	0.00%	66.67%
Cr	0.409	11.2	33.33%	73.33%	45.83%
Ca	0.487	10.3	24.24%	93.33%	45.83%
P	0.406	3.8	84.85%	20.00%	64.58%
Protein-bound solutes					
PCS	0.497	53.8	15.15%	100.00%	41.67%
IS	0.398	21.1	93.94%	13.33%	68.75%
Homocysteine	0.571	27.97	65.63%	57.14%	63.04%
Middle molecules					
IL-1β	0.405	16.41	3.03%	100.00%	33.33%
IL-6	0.393	2.49	81.82%	26.67%	64.58%
IL-18	0.439	99.6	60.61%	46.67%	56.25%
TNF-α	0.481	53.5	9.09%	100.00%	37.50%
iPTH	0.464	54.4	87.50%	20.00%	65.96%
Beta-2-microglobulin	0.504	29040	40.63%	73.33%	51.06%
Molecular markers of nerve injury					
NSE	0.565	2418.23	51.52%	73.33%	58.33%
HSP 70	0.477	0.06	96.97%	13.33%	70.83%
S100B	0.477	227.27	18.18%	93.33%	41.67%
MicroRNA					
miR-134	0.501	1.22	40.63%	73.33%	51.06%
miR-182	0.483	0.02	93.75%	14.29%	69.57%
miR-451	0.503	0.93	69.70%	46.67%	62.50%
miR-486	0.614	32.68	78.79%	53.33%	70.83%

Cumulative ROC analyses for cognitive impairment (CI) prediction in patients receiving dialysis.

**Table 3:** Cumulated top-ranked predictors using ROC analysis.

Cumulated Top-Ranked Variables *^,1^	Variable	Cumulative AUC	Standard Error	95% Confidence Interval
2	Hb and Age	0.837	0.065	0.71–0.965
3	Above plus miR-486	0.897	0.047	0.806–0.988
4	Above plus Gender	0.874	0.054	0.768–0.981
5	Above plus Homocysteine	0.835	0.070	0.698–0.971
6	Above plus NSE	0.848	0.063	0.725–0.971
7	Above plus Education level	0.828	0.064	0.702–0.954
8	Above plus Beta-2-microglobulin	0.824	0.065	0.697–0.951
9	Above plus Albumin	0.827	0.068	0.694–0.959
10	Above plus miR-451	0.798	0.072	0.657–0.939
11	Above plus miR-134	0.794	0.076	0.644–0.943
12	Above plus PCS	0.799	0.075	0.652–0.946
13	Above plus Ca	0.819	0.070	0.682–0.955
14	Above plus miR-182	0.800	0.073	0.658–0.943
15	Above plus TNF-α	0.806	0.071	0.666–0.945
16	Above plus HSP 70	0.799	0.074	0.655–0.943
17	Above plus S100B	0.800	0.075	0.653–0.947
18	Above plus ADMA	0.815	0.073	0.671–0.958
19	Above plus Kt/V	0.823	0.073	0.681–0.965
20	Above plus iPTH	0.833	0.073	0.69–0.976
21	Above plus IL-18	0.835	0.068	0.701–0.968
22	Above plus Cr	0.810	0.074	0.664–0.955
23	Above plus P	0.812	0.075	0.666–0.958
24	Above plus IL-1β	0.812	0.075	0.666–0.958
25	Above plus IS	0.804	0.075	0.657–0.951
26	Above plus IL-6	0.808	0.074	0.662–0.954
27	Above plus 8OHDG	0.808	0.074	0.662–0.954
28	Above plus BUN	0.804	0.075	0.658–0.951

* The sequential of variable was depends on the value of individual area under the curve (AUC).

**Table 4:** Cut-off point of cumulated risk score identified by ROC analysis.

Number of Dichotomized Variables *	Sensitivity (95% CI)	Specificity (95% CI)	Youden’s Index	Correctly Classified	LR+ (95% CI)	LR− (95% CI)
S1	100% (89.4%-100%)	26.67% (7.79%-55.1%)	26.67%	77.08%	1.36 (1.01–1.85)	-
S2	81.82% (64.5%-93%)	86.67% (59.5%-98.3%)	68.49%	83.33%	6.14 (1.67–22.5)	0.21 (0.1–0.44)
S3	27.27% (13.3%-45.5%)	100% (78.2%-100%)	27.27%	50.00%	-	0.73 (0.59–0.9)

Abbreviations: LR+, likelihood ratio for a positive test result; LR−, likelihood ratio for a negative test result. * The number of dichotomized variables was the cumulated top-ranked predictors from Table 3, including Hb, age, and miR-486. S1: Patients have any one of the following characteristics: Hb ≥ 10.7 g/dL, age ≥ 63 years, and miR-486 ≥ 32.68. S2: Patients have any two of the following characteristics: Hb ≥ 10.7 g/dL, age ≥ 63 years, and miR-486 ≥ 32.68. S3: Patients have all of the following characteristics: Hb ≥ 10.7 g/dL, age ≥ 63 years, and miR-486 ≥ 32.68.

**Table 5:** Relationship between cumulated risk score and selected variables. (*n* = 48).

Cumulated Risk Score	Total	Hb ≥ 10.7		Age ≥ 63		miR-486 ≥ 32.68	
		(*n* = 21)		(*n* = 28)		(*n* = 33)	
	*n*	*n*	%	*n*	%	*n*	%
S0	4	-	-	-	-	-	-
S1	15	2	13.3	3	20.0	10	66.7
S2	20	10	50.0	16	80.0	14	70.0
S3	9	9	100.0	9	100.0	9	100.0

S0: Patients have none of the following characteristics: Hb ≥ 10.7 g/dL, age ≥ 63 years, and miR-486 ≥ 32.68. S1: Patients have any one of the following characteristics: Hb ≥ 10.7 g/dL, age ≥ 63 years, and miR-486 ≥ 32.68. S2: Patients have any two of the following characteristics: Hb ≥ 10.7 g/dL, age ≥ 63 years, and miR-486 ≥ 32.68. S3: Patients have all of the following characteristics: Hb ≥ 10.7 g/dL, age ≥ 63 years, and miR-486 ≥ 32.68.

**Table 6:** Logistic regression for mild cognitive impairment in patients receiving hemodialysis.

Variable	OR (95%CI)	*p*
**Univariate**		
Hb	2.29 (1.13–4.64)	0.022
Age	1.06 (0.99–1.13)	0.106
miR-486	4.24 (1.14–15.79)	0.031
**Multivariate**		
Hb	2.74 (1.13–6.67)	0.026
Age	1.04 (0.96–1.13)	0.351
miR-486	7.54 (1.47–38.6)	0.015
**2-Order Interaction**		
Hb*Age	1.01 (1.001–1.01)	0.019
Hb*miR-486	1.17 (1.03–1.32)	0.016
Age*miR-486	1.03 (1.004–1.05)	0.019
**3-Order Interaction**		
Hb*Age*miR-486	Omitted	-

## Supplementary Materials

The following are available online at https://www.mdpi.com/2073-4409/9/10/2303/s1, Figure S1: ROC curve of best cut-off point for cumulated top-three ranked variables for MMSE category derived from CDR dichotomous results, Table S1: The distribution of MMSE in different CDR group, Table S2: Cut-off point of cumulated risk score identified by ROC analysis for MMSE category derived from CDR dichotomous results.
